# The DNA hypermethylation phenotype of colorectal cancer liver metastases resembles that of the primary colorectal cancers

**DOI:** 10.1186/s12885-020-06777-6

**Published:** 2020-04-06

**Authors:** Stephany Orjuela, Mirco Menigatti, Peter Schraml, Patryk Kambakamba, Mark D. Robinson, Giancarlo Marra

**Affiliations:** 1 Institute of Molecular Cancer Research, University of Zurich, Winterthurerstrasse 190, 8057 Zurich, Switzerland; 2 Institute of Molecular Life Sciences, University of Zurich and SIB Swiss Institute of Bioinformatics, Zürich, Switzerland; 3 Department of Pathology and Molecular Pathology, University of Zurich, Zürich, Switzerland; 4 Division of Surgical Research, University of Zurich, Zürich, Switzerland

**Keywords:** Normal colorectal mucosa, Colorectal cancer, Liver metastasis, DNA methylation, CpG sites, CpG islands, Methyl-binding domain, MBD capture, Differentially methylated regions

## Abstract

**Background:**

Identifying molecular differences between primary and metastatic colorectal cancers—now possible with the aid of omics technologies—can improve our understanding of the biological mechanisms of cancer progression and facilitate the discovery of novel treatments for late-stage cancer. We compared the DNA methylomes of primary colorectal cancers (CRCs) and CRC metastases to the liver. Laser microdissection was used to obtain epithelial tissue (10 to 25 × 10^6^ μm^2^) from sections of fresh-frozen samples of primary CRCs (*n* = 6), CRC liver metastases (*n* = 12), and normal colon mucosa (*n* = 3). DNA extracted from tissues was enriched for methylated sequences with a methylCpG binding domain (MBD) polypeptide-based protocol and subjected to deep sequencing. The performance of this protocol was compared with that of targeted enrichment for bisulfite sequencing used in a previous study of ours.

**Results:**

MBD enrichment captured a total of 322,551 genomic regions (249.5 Mb or ~ 7.8% of the human genome), which included over seven million CpG sites. A few of these regions were differentially methylated at an expected false discovery rate (FDR) of 5% in neoplastic tissues (primaries: 0.67%, i.e., 2155 regions containing 279,441 CpG sites; liver metastases: 1%, i.e., 3223 regions containing 312,723 CpG sites) as compared with normal mucosa samples. Most of the differentially methylated regions (DMRs; 94% in primaries; 70% in metastases) were *hyper*methylated, and almost 80% of these (1882 of 2396) were present in both lesion types. At 5% FDR, no DMRs were detected in liver metastases vs. primary CRC. However, short regions of low-magnitude *hypo*methylation were frequent in metastases but rare in primaries. Hypermethylated DMRs were far more abundant in sequences classified as intragenic, gene-regulatory, or CpG shelves-shores-island segments, whereas hypomethylated DMRs were equally represented in extragenic (mainly, open-sea) and intragenic (mainly, gene bodies) sequences of the genome. Compared with targeted enrichment, MBD capture provided a better picture of the extension of CRC-associated DNA hypermethylation but was less powerful for identifying hypomethylation.

**Conclusions:**

Our findings demonstrate that the hypermethylation phenotype in CRC liver metastases remains similar to that of the primary tumor, whereas CRC-associated DNA hypomethylation probably undergoes further progression after the cancer cells have migrated to the liver.

## Background

Among secondary tumors of the liver, metastases from colorectal adenocarcinomas are the easiest to identify histopathologically. In most cases, they exhibit glands strongly resembling those of the primary colorectal cancers (CRCs), their lumens lined by tall columnar cells and filled with necrotic debris (“dirty necrosis”) [1]. The histopathological grade and differentiation capacity of a primary colorectal cancers can be even recapitulated in xenografted tumor organoids [2]. Given the strikingly different environments in which the primary and metastatic (or xenografted) tumor cells are forced to grow, this phenotypic robustness is remarkable. It might reflect the existence of a genetic–epigenetic program that remains more or less unchanged, even when the CRC cells migrate to the liver and establish a new tumor there. This hypothesis is consistent with reports of high genomic concordance between some primary CRCs and their metastases [3, 4] and with claims that the DNA-methylation-based epigenetic profile of liver metastases of unknown origin can reliably reveal the lesions’ primary cancer source [5].

However, the genetic and epigenetic profiles of the CRC liver metastases will never be 100% identical to those of the primary tumor growing in the gut. Among other things, some anticancer drugs cause genetic and/or epigenetic changes that favor the selection and emergence of drug-resistant cellular clones [6]. Some degree of divergence from the primary tumor is thus inevitable, and its magnitude is probably characterized by substantial inter-tumor and inter-tumor-type variation [7, 8]. Investigation of this divergence requires powerful omics tools capable of exploring most if not all of the human genome.

Genome-wide analysis of DNA methylation, for example, can be particularly informative in this setting. During embryogenesis, the DNA methylome undergoes profound remodeling, with the removal and addition of methyl groups at cytosine bases, primarily those making up CpG dinucleotides. These changes are associated with markedly modified gene-expression (although the mechanisms underlying this association are still incompletely understood) [9–13], and play pivotal roles in the processes of cellular differentiation and organ specification [14, 15]. Once the large intestine methylome is established, however, it is chemically stable and faithfully maintained by mitotic inheritance for the life of the individual.

A major exception to this rule occurs with the onset of neoplasia in the gut. During colorectal tumorigenesis (as well as that occurring in other organs), many normally unmethylated CpG islands located in gene promoters become heavily methylated. For a few of these promoters, the hypermethylation has been strongly linked to gene silencing with potentially crucial roles in tumorigenesis. Paradigmatic is the epigenetic inactivation of the DNA mismatch repair gene *MLH1*, which leads to the emergence of post-replicative DNA mutations and microsatellite instability (MSI) [16]. The hypermutator phenotype of these cancers has distinctive effects on their prognosis and their sensitivity to chemotherapy [17–19]. They account for about 15% of all CRCs, and are presently classified as consensus molecular subtype 1 in the gene-expression-based CRC classification [20] and as MSI/CIMP-H (CpG island methylator phenotype-high) in the TCGA classification of gastrointestinal cancers [21].

We recently investigated the DNA methylomes of precancerous and cancerous colorectal lesions [13]. In addition to our extensive characterization of the *hyper*methylation phenotype of gene regulatory genomic regions in both types of tumor, we also confirmed the presence in these lesions of another classical tumor-associated change in the methylome: widespread *hypo*methylation. These findings were obtained with bisulfite sequencing of DNA subjected to targeted enrichment for regions containing CpG islands since whole-genome sequencing is associated with well-known constraints in terms of costs, data storage, and analysis. In the present study, we used methyl-CpG-binding domain (MBD) sequencing to explore the methylomes of normal and neoplastic colon tissues, with the aim of discovering whether the process of colorectal tumor-associated epigenetic remodeling continues to evolve in CRC cells that have migrated to and established metastases in the liver. We also compared the performances of the MBD- and targeted-enrichment methods in the characterization of the CRC methylome.

## Methods

### Tissues

Tissues were prospectively collected at the University Hospital of Zurich (Switzerland) with institutional research ethics committee approval. Donors provided written consent to tissue collection, testing, and data publication. Samples were numerically coded to protect donors’ rights to confidentiality.

Immediately after resection, samples of normal colon mucosa, primary CRCs, and CRC liver metastases were frozen in liquid nitrogen and stored at − 80 °C. They included six primary tumors, three of which were accompanied by patient-matched samples of normal mucosa from the same gut segment (> 2 cm from the tumor), and twelve liver metastases (none of which were from the primary CRC donors) (Table 1). Nine patients were diagnosed with a single metastasis. For the three who developed multiple metastases, the largest lesion was included in the study. All of the primary and secondary lesions were microsatellite-stable and CpG island methylator phenotype-negative.

**Table 1:** Characteristics of tissues included in the study

Patient number	Tissue	Site	Lesion size (mm)	Stage and grade of primary cancer ^a^
C1c	primary CRC	R	30 × 25	T4aN2bR0cM1 / G2
C1n	normal mucosa	R		
C2c	primary CRC	S	27 × 10	T3N0M0R0 / G3
C2n	normal mucosa	S		
C3c	primary CRC	R	25 × 25	T2N1bM0 / G2
C3n	normal mucosa	R		
C4	primary CRC	T	18 × 15	T4aN0M0R0 / G2
C5	primary CRC	S	50 × 20	T3N2acM1 / G2
C6	primary CRC	A	30 × 25	T4aN2bcM1 / G3
M1	CRC metastasis	liver	13 × 7	pT3pN2M0 / G3
M2	CRC metastasis	liver	25 × 13	pT4pN0M0 / G3
M3	CRC metastasis	liver	10 × 6	pT3pN0cM1 / G3
M4	CRC metastasis	liver	20 × 7	pT1pN1M0 / G2
M5	CRC metastasis	liver	15 × 10	pT4pN1M1 / G2
M6	CRC metastasis	liver	10 × 5	pT3N0M0 / G2
M7	CRC metastasis	liver	15 × 15	pT1pN1M0 / G2
M8 ^b^	CRC metastasis	liver	10 × 8	pT3N1M0 / G2
M9 ^b^	CRC metastasis	liver	20 × 20	pT3N0M0 / G2
M10 ^b^	CRC metastasis	liver	25 × 15	pT3N2M0 / G3
M11 ^b^	CRC metastasis	liver	10 × 8	pT3pN1M0 / G2
M12 ^b^	CRC metastasis	liver	15 × 8	pT3pN1M0 / G2

Abbreviations: *CRC* colorectal cancer, *R* rectum, *S* sigmoid, *T* transverse colon, *A* ascending colon

^a^ Cancer TNM and grade classification in Sobin LH, Wittekind C. TNM classification of malignant tumors. 6th ed. New York, NY: Wiley-Liss, 2002.

^b^ Patients who received chemotherapy 1–20 months before resection of the liver metastasis. All other donors were chemo-naïve when sampled tumors were resected

### Laser tissue microdissection

Laser tissue microdissection was done with a CellCut system (Molecular Machines & Industries, Glattbrugg, Switzerland). Briefly, 10 μm-thick sections were cut with a Hyrax C60 cryostat (Zeiss, Feldbach, Switzerland) from frozen tissues embedded in Tissue-Tek O.C.T. (i.e., optimum cutting temperature formulation; Sakura, Alphen aan den Rijn, The Netherlands). The sections were placed on membrane slides (Molecular Machines & Industries), fixed in propanol for 45 s, and covered with one drop of Mayer’s hematoxylin (MHS128, Sigma-Aldrich, Buchs, Switzerland) for 45 s. After vigorous washing, the sections were sequentially immersed in 0.1% ammonia (10 s), propanol (45 s), and xylene (45 s) and dried for 5 min. Stained tissues were then subjected to laser microdissection using special tubes with caps to which the dissected sections adhered (Molecular Machines & Industries, Glattbrugg, Switzerland). Epithelial cells from normal or neoplastic crypts were selectively collected on the cap, with care taken to minimize stromal-cell contamination. Between 10 and 25 × 10^6^ μm^2^ of dissected epithelium (= ≈ 100,000 to 250,000 epithelial cells) was collected from each sample. Immediately after dissection, DNA was extracted with the QIAmp DNA Micro kit (Qiagen, Hombrechtikon, Switzerland) and quantified with a Qubit fluorometer and dsDNA HS Assay kit (Thermo Fisher Scientific, Reinach, Switzerland) (total yield: 100–500 ng DNA).

### MBD-peptide-based capture of DNA for deep sequencing

Methylated DNA for sequencing was isolated using an MBD-capture protocol [22]. Reaction volumes were scaled down to successfully process the small volumes of genomic DNA obtained with laser tissue microdissection. Briefly, 100 ng of input DNA was fragmented to an average length of 200 bp in a Covaris (Brighton, UK) S2 ultrasonicator. Recombinant MBD2 protein-mediated enrichment (MBDE) for methylated DNA was carried out with the MethylMiner kit (ThermoFisher, Waltham, MA, USA) using a single elution step with 2000 mM NaCl elution buffer. Multiplex Illumina libraries were prepared with the NEBNext Ultra DNA Library Prep Kit (New England Biolabs, Ipswich, MA, USA), and their sizes and concentrations were evaluated with the Agilent (Santa Clara, CA, USA) 2200 TapeStation system. Libraries were sequenced on an Illumina 2500 system (Illumina, San Diego, CA, USA) (on average 50-bp single-end reads, 40 million reads per sample).

Raw methylome data are deposited in *ArrayExpress* (accession number: E-MTAB-8232).

### Detection of differential methylation

MBD-sequencing reads were aligned to the GRCh37/hg19 human reference genome using bwa (version 0.7.12-r1039) and the BWA-MEM algorithm [23] and de-duplicated with Picard (Picard Toolkit, version 2.13.2; 2018. Broad Institute, GitHub Repository: http://broadinstitute.github.io/picard/).

The R-package csaw (version 1.14.1) [24] was used to identify regions that were differentially methylated in neoplastic tissues (primary and/or metastatic) vs. normal mucosa or in metastatic vs. primary cancers. The number of reads per sample was counted in consecutive overlapping windows (length: 200-bp, overlap: 190 bp). Windows with minimum count sums of 30 across samples were kept. Additional filtering was used to exclude windows with average log counts per million that were below − 1. Reads aligned to the X or Y chromosome were excluded from analysis. The csaw package uses methods from edgeR (version 3.22.3) to identify differential binding [25] (i.e., NB GLM model to fit read counts). Each filtered window was tested for differential methylation (i.e., differential binding of the methyl-binding protein) associated with disease status (CRC, primary or metastatic, vs. normal mucosa) or disease stage (primary vs. metastatic CRC). After testing, windows separated by no more than 50 bp were merged to form regions, and *P-*values were calculated for each region using the Simes method, as described in the package. Differentially methylated regions (DMRs) were classified as *hyper*methylated or *hypo*methylated based on the direction of the methylation change in the majority of windows included in the region.

**Fig. 1:**
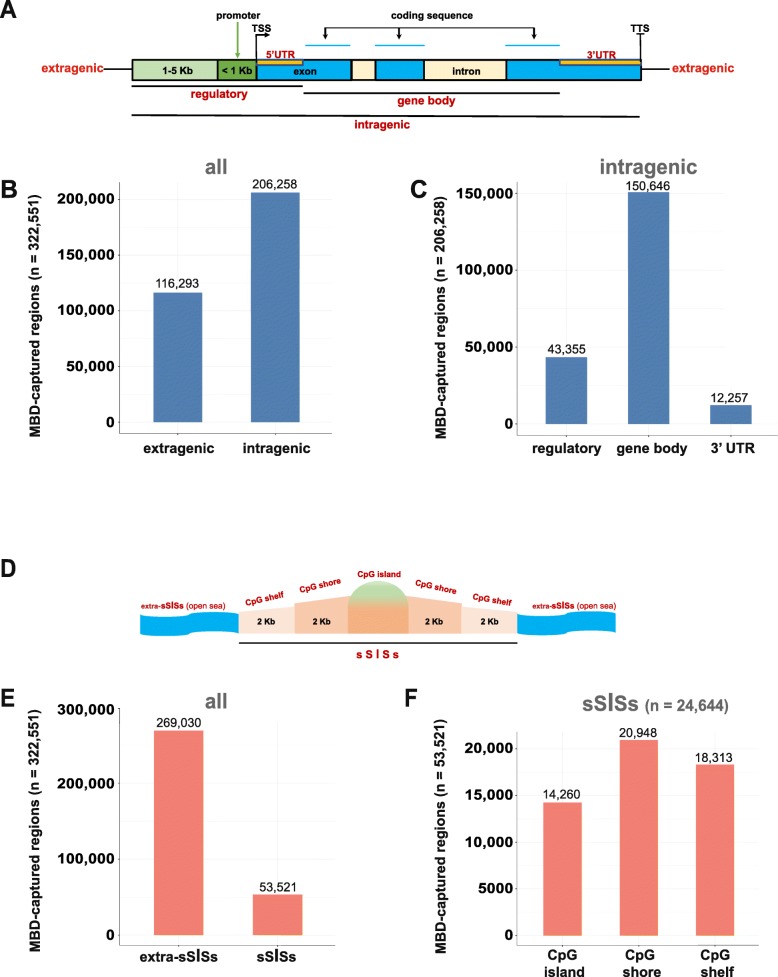
Genomic distribution and functional classification of MBD-captured regions within the genome. **a**. Schematic showing components of an "intragenic" genomic region. Areas outside such regions were classified as "extragenic". **b**. MBD-captured regions (total: 322,551) classified as extragenic vs. intragenic. **c**. Distribution of the 206,258 intragenic MBD-captured regions by subregions: *gene regulatory* (including 5'UTRs and regions 5 kb upstream from the TSS); *gene body* (including exonic and intronic sequences between the end of the 5'UTR and the beginning of the 3'UTR); and the *3'UTRs*. **d**. Schematic showing components of an “sSISs” region (i.e., a CpG island and flanking CpG shores and shelves). Areas outside such regions were defined "extra-sSISs". Schematics in this panel and in panel A were adapted from diagrams contained in the R-package *annotatr* vignette*.***e**. MBD-captured regions classified as sSISs vs. extra-sSISs. **f**. Distribution of the 53,521 sSISs MBD-captured regions by subregions (CpG islands, shores, and shelves). N.B. After exclusion of the X and Y chromosomes, the human genome contains 26,361 canonical CpG *islands*, 15,423 of which were covered by MBDE. (See Discussion).

Regions were annotated using the annotatr R-package [26], which overlays regions of interest with predefined annotations from external sources. Annotations were grouped to create three “intragenic” categories: 1) regulatory: genomic regions 5 kb upstream from the TSS, and 5’UTRs; 2) gene body: exons and introns (from the end of the 5’UTR to the beginning of the 3’UTR); and 3) the 3’UTRs. Everything outside these regions was considered “extragenic” (Fig. 1A). Regions were also classified based on their CpG density: CpG islands and the CpG shores and shelves flanking them were grouped together and referred to as an sSISs (shelf–shore-island-shore-shelf) region, and everything outside these regions was classified as extra-sSISs (Fig. 1D). These regions were overlaid onto all windows tested for differential methylation.

Visualization was performed using the ggplot2 (version 3.0.0) [27] and UpSet (version 1.3.3) [28] R-packages. Analyses were performed with R versions 3.6.0.

### Estimated CpG density

Each CpG site (CpGs) (Team TBD 2014; BSgenome.Hsapiens.UCSC.hg19; R package version 1.4.0.) was centered within a 200-bp window, and the ratio of the observed to expected numbers of CpGs (O:E CpG ratio) [29] was calculated for each window, as a measure of its CpG density (low - O:E CpG ratios < 0.3; intermediate > = 0.3 but < 0.6; high > = 0.6 – high density).

### Comparison of MBDE- and targeted bisulfite-sequencing in colorectal cancer

In a previous study [13], we used targeted bisulfite sequencing (SeqCapEpi CpGiant protocol, Roche, Basel, Switzerland) to assess DNA methylation in three CRCs (all microsatellite-stable and CpG island methylator phenotype-negative) and matched samples of normal mucosa (*ArrayExpress* accession number: E-MTAB-6949). The results of those experiments were compared with those obtained in the present study with MBD-capture sequencing, to identify potential strengths and weaknesses of the two enrichment methods for CRC methylome characterization.

For this analysis, we considered CpGs covered by the targeted enrichment (TE) procedure, those covered by MBD enrichment (MBDE), and those covered by both enrichment methods. Because nearby CpGs are frequently co-methylated [30, 31], the log-fold change (logFC) and *P*-value for each CpG site in a given MBD-captured region were assumed to be identical to those calculated for the region as a whole. For CpGs covered by the TE procedure, differences in methylation proportions and *P*-values were calculated with the BiSeq R package (Hebestreit K, Klein H, 2018*. BiSeq: Processing and analyzing bisulfite sequencing data.* R package version 1.22.0), by modeling the methylation level within a beta regression and estimating the group effect, in this case primary CRC vs. normal tissue.

CpGs covered by both methods were classified according to the CpG density of the region in which they were located (as specified above) and the direction of the differential methylation identified at the site in primary CRCs (vs. normal mucosa): *hyper*methylated (TE: difference in methylation proportions > 0; MBDE: logFC > 0 and *P*-value < 0.05); *hypo*methylated (TE: methylation proportion difference < 0; MBDE: logFC < 0 and *P*-value < 0.05); *iso*methylated (TE: methylation proportion difference = 0; MBDE: logFC = 0 and *P*-value = > 0.05). The strength of the linear association between the two methods was calculated with the Pearson correlation coefficient.

DMRs identified from TE data with the BiSeq R package were overlaid with DMRs detected from MBDE reads, and regions were classified as overlapping if they shared a sequence of at least 1 bp.

Code implemented in the analysis is available at https://github.com/sorjuela/livermetastasis_MBDseq_paper.

## Results

DNA extracted from the 21 laser-microdissected, colorectal tissue samples was subjected to MBD-capture sequencing (Table 1 and Supplementary Fig. 1 in Additional Files). Fig. 1 shows the genomic locations of the 322,551 methylated regions isolated with this technology. Most (64%) were located in the “intragenic” genome, which contains coding genes, their regulatory segments, and 3’UTRs (Fig. 1a and b), and 73% of these intragenic regions were situated within a gene body (Fig. 1c). As for CpG statuses (Fig. 1d), 17% of all the captured regions were located in sSISs sequences (Fig. 1e), predominantly (73%) in CpG shores or shelves rather than islands (Fig. 1e).

**Fig. 2:**
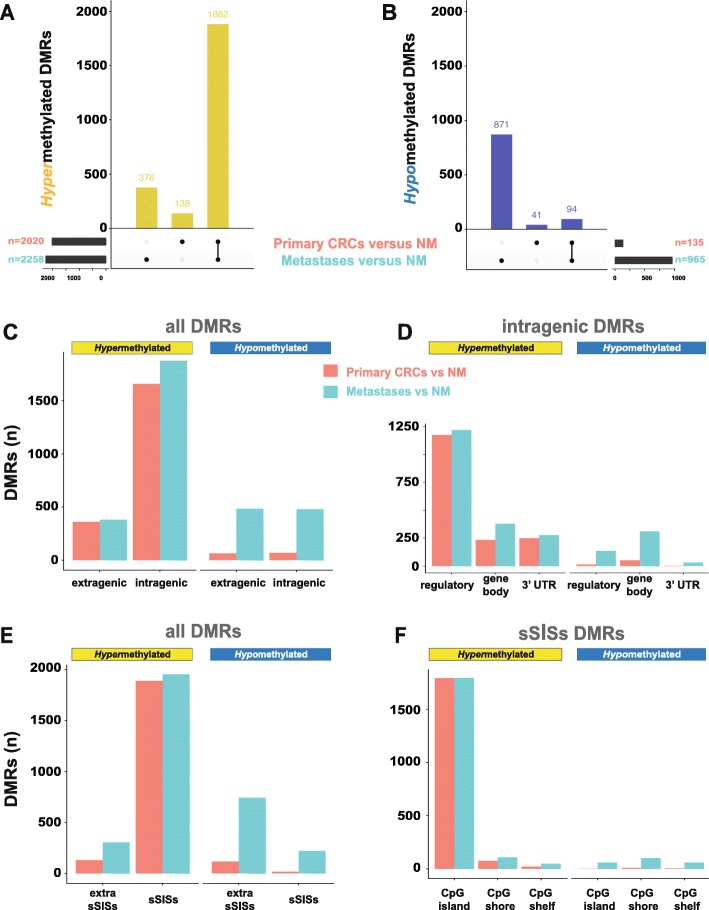
Genomic regions in primary CRCs or CRC liver metastases displaying differential methylation relative to that in normal colon mucosa (NM). **a** and **b**. Differentially methylated regions (DMRs) characterized by *hyper*methylation and *hypo*methylation (vs. NM) present in primary CRCs, metastatic CRCs, or both. **c**, **d**, **e**, and **f**. Distributions of *hyper*methylated and *hypo*methylated DMRs in the extragenic vs. intragenic genomes; among the intragenic genome components; between the sSISs and extra-sSISs genomic segments, and among the sSISs components, respectively. (See Figure 1 for topography of genomic segments.)

Few of the 322,551 MBD-captured regions displayed significant differential methylation (defined by an adjusted *P*-value cutoff of 0.05) in the cancer tissues (*n* = 2155 in the primary CRCs; *n* = 3223 in the liver metastases) in comparison with normal mucosal samples (Fig. 2, Supplementary Table 1 in Additional file 5), and no DMRs were identified when primary and metastatic cancers were compared. Five of the 12 liver metastases came from patients who had received chemotherapy 1–20 months before liver resection (Table 1). No significant differences were found between their methylomes and those of the seven metastases from chemo-naïve patients. For this reason, all 12 metastatic lesions were considered as a single group in the statistical analysis.

The vast majority of DMRs identified in tumor tissues (94% of those in the primaries, 70% in metastases) were *hyper*methylated relative to the normal mucosa. In contrast, *hypo*methylated DMRs were generally less common, and almost all of them were found in liver metastases rather than in the primary tumors (Fig. 2a and b). Hypomethylated DMRs were thus a distinctive feature of the metastatic lesions (Fig. 2b), whereas most hypermethylated DMRs were found in both types of neoplastic tissue (Fig. 2a). In both primary CRCs and metastases, hypermethylated DMRs were far more abundant in intragenic than extragenic regions (Fig. 2c), in regulatory segments than in gene bodies or 3’UTRs (Fig. 2d), in sSISs than in extra-sSISs segments (Fig. 2e), and in CpG islands than in CpG shores/shelves (Fig. 2f). In contrast, hypomethylated DMRs were equally represented in the extragenic and intragenic genomes (Fig. 2c). Within intragenic regions, they were more frequent in gene bodies (than in regulatory segments or 3’UTRs) (Fig. 2d). Hypomethylation was more common in the extra-sSISs genome (Fig. 2e), but those located within sSISs areas displayed similar frequencies in CpG islands, shores, and shelves (Fig. 2f). The distribution of DMR lengths among the genomic segments discussed above is shown in the Additional Files (Supplementary Fig. 2).

**Fig. 3:**
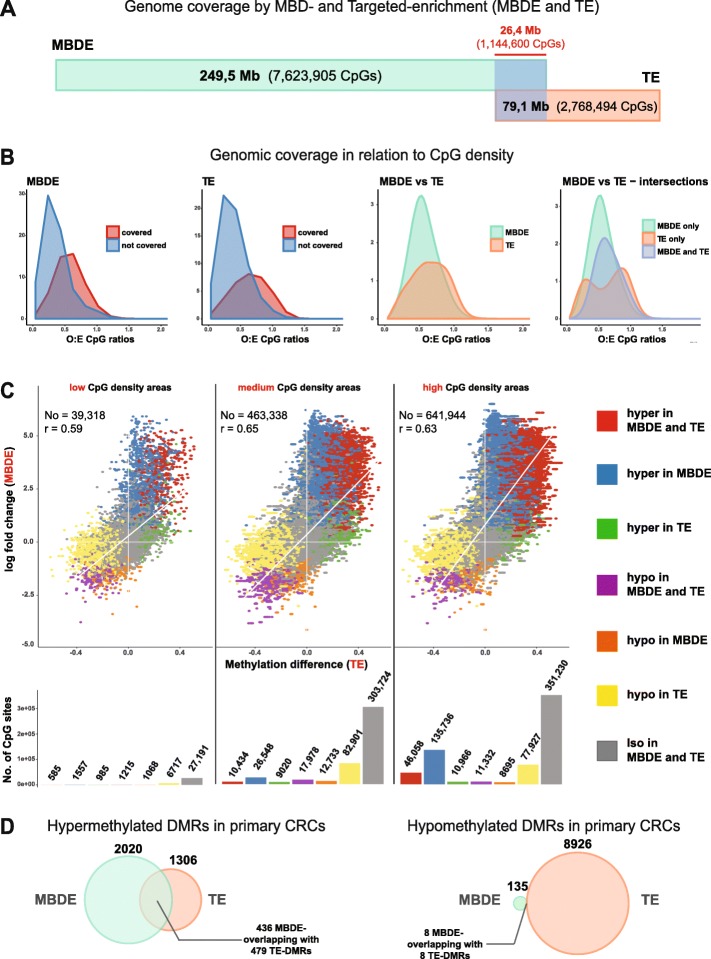
Performance of MBD enrichment (MBDE) and targeted enrichment (TE) in identifying differentially methylated CpGs or regions (DMRs) in primary CRCs (vs. normal mucosa). **a**. Compared with TE, MBDE captured 3 times more genomic base pairs (including 33.4% of TE-captured base pairs) and almost 3 times more CpGs (including 41.3% of TE-captured sites). **b**. First three density plots, from left: Compared with CpGs missed by both methods (mean O:E CpG ratios: 0.35 for MBDE, 0.39 for TE), the CpGs captured by each method were preferentially located in genomic areas with relatively high CpG densities (mean O:E CpG ratios: 0.58 for MBDE, 0.65 with TE). Fourth density plot: CpGs captured by MBDE only, by TE only or by both methods tended to be located in genomic areas with similar CpG densities (mean O:E CpG ratios: 0.56 for MBDE, 0.65 for TE, 0.66 for both methods). **c**. Methylation changes (log fold change in MBDE, beta-values in TE) at CpGs captured by both methods in low-, medium-, and high-CpG-density areas. (See *Methods* for calculation of differential methylation with each method and for calculation and classification of CpG density.) Intermethod correlation values are shown for each density area. For most of the CpGs captured by both methods (gray bars), no methylation differences in primary CRCs (vs. normal mucosa) were identified with either MBDE or TE. The number of hypermethylated CpGs detected by both methods (red bars) steadily increased with increasing CpG rates. In addition, in medium- and high-CpG-rate areas of the genome, MBDE captured hypermethylated CpGs that were not found to be significantly hypermethylated with TE (blue vs. green bars). Hypomethylated CpGs were more frequently identified by TE than MBDE (yellow vs. orange bars). **d**. The CpG site trends were confirmed by analysis of DMRs. Over 400 hypermethylated DMRs were identified with both methods (in addition to the 1584 identified only by MBDE and the 827 detected only with TE). However, the overlap of commonly identified hypermethylated DMRs is likely underestimated because of the use of different analytical packages to identify them with MBDE or TE (see example in Supplementary Figure 3 C). Hypomethylated DMRs were in contrast identified almost exclusively by TE (examples in Supplementary Figure 3 D and E).

As detailed in the Methods section, we then compared the primary CRC methylomes characterized with MBDE with the results obtained in other primary CRCs using TE [13]. As shown in Fig. 3a, MBDE covered a larger portion of the genome with a higher CpG content (249.5 Mb containing ~ 7,6 million CpGs vs. ~ 79.1 Mb with ~ 2.8 million CpGs with TE), and around 1,1 million CpGs were captured by both methods. Both methods preferentially covered CpGs in regions where the density of these dinucleotides was relatively high (Fig. 3b), and 1,105,282 (96.5%) of the CpGs covered by both methods were located in regions with an intermediate- or high-CpG density (Fig. 3c).

Cancer-associated changes in methylation levels identified with the MBDE and TE displayed a moderately strong positive correlation (*r*: from 0.59 to 0.65, Fig. 3c, upper panel). However, the methods differed substantially in calling hyper- or hypomethylation at individual CpGs (Fig. 3c, lower panel). Of the 1,144,600 CpGs covered by both methods, 57,077 (5%) were concordantly identified as hypermethylated and 30,525 (2.7%) as hypomethylated. More frequently, however, the two methods yielded discordant results: indeed, 163,841 (14.3%) of the CpGs were identified as significantly hypermethylated only with MBDE, and a similar proportion (*n* = 167,545 [14.6%]) emerged as significantly hypomethylated only with TE. Accordingly, hypermethylated DMRs were more frequently identified in primary CRCs with MBDE than with TE, whereas hypomethylated DMRs were almost exclusively identified with TE (Fig. 3d, and examples of DMRs in Additional Files, Supplementary Fig. 3). The lower number of hypermethylated DMRs identified with TE was due in part due to the lack of SeqCapEpi CpGiant probes targeting many of the regions found to be hypermethylated with MBDE (Supplementary Fig. 3B) and in part to the different computational approaches used to analyze the MBDE and TE data sets (Supplementary Fig. 3C). As for the difference involving hypomethylated DMRs, it probably reflects the fact that these regions were generally shorter than their hypermethylated counterparts and displayed less markedly altered methylation levels (hypo beta-value mean: 0.21, hyper beta-value mean: 0.31; see also Supplementary Table 1, Supplementary Figs. 2 and 3 D-E, and Fig. 1e of our previous study [13]).

## Discussion

We used a high-stringency MBD-protein-based enrichment protocol to obtain methylated DNA for deep sequencing from CRCs (primary and liver metastases) and normal colon mucosa. The portion of the genome sequenced (7.8%) included ~ 27% of the ~ 28 million CpGs found therein (~ 7.6 × 10^6^). Small fractions of the MBD-isolated regions were differentially methylated in primary (2155 regions including 279,441 CpGs) or metastatic CRC (3223 regions containing 312,723 CpGs) samples relative to the unmatched samples of normal colon mucosa we tested. Importantly, the *hyper*methylation phenotype of the liver metastases closely resembled that of primary CRCs, in terms of both the identity and location of the hypermethylated DMRs. We have previously shown that this phenotype is already evident in precancerous colorectal lesions (i.e., sessile serrated lesions and, to a lesser extent, adenomatous polyps), and it becomes increasingly obvious in CRCs [13]. Our present findings suggest that this progression reaches a plateau before CRC cells seed the liver. In contrast, the spread of CRC-associated *hypo*methylation continues after the tumor cells metastasize to the liver. The extent of this progression requires further investigation with whole-genome analysis of the methylome (see below), but it is tempting to speculate that late increases in hypomethylation might contribute to metastasis-specific alterations in the gene expression, genomic stability, and/or drug susceptibility of CRCs [32–36].

Our study is the first to use genome-wide deep-sequencing to compare the methylomes of primary CRCs and CRC liver metastases. To our knowledge, only three previous studies [37–39] have analyzed this issue at the genome-wide level. In two studies, freshly collected, patient-matched tissue pairs (nine in one case [37], three in the other [38]) were analyzed using a methylated CpG island amplification microarray approach involving methylation-specific, restriction-enzyme digestion of defined CpGs in approximately 6000 gene promoters [40, 41]. Both found that the hypermethylation phenotypes of CRC liver metastases closely resembled those of their primary cancer counterparts, leading the investigators to conclude —as we have— that most of the DNA hypermethylation associated with colorectal tumorigenesis probably occurs *before* the disease spreads to the liver. Our in silico analysis of previously published llumina Infinium 450 microarray data on six patient-matched tissue pairs [39] also confirmed that the methylomes of primary and metastatic colorectal cancers are similar (Additional Files, Supplementary Fig. 4). This conclusion is also supported by findings of a recent analysis of 70 pairs of formalin-fixed, paraffin-embedded tissues, which revealed concordance between primary CRCs and matched metastases taken from different organs in the CpG island hypermethylation phenotype at five gene promoters [42]. The tissues we tested were prospectively collected to obtain, from fresh samples, high-quality DNA for MBD capture, and this markedly reduced our chances of obtaining matched samples of colorectal cancers and their corresponding liver metastases in the timeframe of the study. (The clinical management of patients with colorectal cancer—before and after detection of liver metastases—is usually a fairly long process marked by multiple surgical and chemotherapeutic interventions, often carried out in different hospitals.) Although this is undeniably a limitation, the strikingly similar hypermethylation phenotypes observed in the unmatched primary and metastatic tumors suggests that similar or even greater concordance would probably be evident in matched samples. Our use of laser-capture tissue microdissection probably played a key role in reducing the variability between primary and metastatic epithelial tumors by eliminating normal and tumor-related stromal cells from their respective microenvironments, an important aspect that recently emerged in a gene-expression study of primary and metastatic colorectal cancers [43].

Our findings on hypomethylation are also in agreement with the previous observations of Hur et al. [35], who found hypomethylation of long interspersed nuclear element-1 (LINE-1) sequences in 77 formalin-fixed, paraffin-embedded samples of primary CRCs (vs. normal mucosa), and this alteration was even more evident in matched samples of liver metastases. The fact that some of the hypomethylated LINE-1 sequences were found to be located within the intronic regions of proto-oncogenes whose expression was increased in liver metastases points intriguingly to possible functional consequences of the late increase of hypomethylation in cancer cells seeding in the liver [35].

The present study was conducted exclusively on microsatellite stable/non-CIMP CRCs, which are far more common than CRC with MSI/CIMP-H phenotype. The decision to focus our work on the more frequently encountered phenotype was motivated by the difficulties we encountered in the prospective collection of samples (see above) and the costs of the genome-wide analysis of the DNA methylome. In addition, our previous work [13] has shown that the DNA methylome of primary microsatellite stable/non-CIMP CRCs differs from that of MSI/CIMP-H primaries. Data in the literature are lacking on the possible evolution of the MSI/CIMP-H CRC methylome during metastasis. Genome-wide analysis of the methylome should therefore be extended to this molecular type of CRCs to determine whether hyper−/hypomethylation changes between primary and metastatic tumors are CRC-type specific.

We found no evidence that chemotherapy significantly alters the methylomes of CRC liver metastases. This is an important issue in view of the emergence of drug-resistant clones that might exhibit clinically-relevant epigenomic changes [44, 45], and our finding obviously requires further and more in-depth investigation. The timing of chemotherapy relative to primary and metastatic tumor resections varied widely in our study, as did the drugs administered. All, however, were cytotoxic agents, and it is important to extend the investigation to include the possible effects of more recently introduced targeted approaches, such as anti-EGFR antibodies.

Bisulfite sequencing is still considered the gold standard technique for analyzing DNA methylomes. However, bisulfite conversion of unmethylated cytosines causes substantial DNA damage [46, 47], which can be a major concern when the amount of input DNA extracted from clinical samples is limited (e.g., the laser-microdissected sections of frozen tissues used in our study). Using MBDE, we obtained high-quality methylome data with only 100 ng of input DNA per sample, but reliable results with this enrichment method can reportedly be obtained with volumes as small as 15 ng [48]. Furthermore, owing to cost considerations and computational constraints, bisulfite sequencing analysis is usually limited to a genome-wide *selection* of regions, such as that obtained with the targeted enrichment step we used for our bisulfite-sequencing analysis of the methylomes of normal, precancerous, and cancerous colorectal tissues [13]. Metastatic CRCs were not included in that study, but comparison of the data it generated on primary cancers and normal mucosa with those obtained here provided insights into the pros and cons of the two pre-sequencing enrichment protocols (Fig. 3).

As expected, MBDE covered a larger portion of the genome and more CpG dinucleotides than TE (Fig. 3a). The probes used for TE, which were designed a priori*,* target specific genomic loci consisting mainly of CpG islands in regulatory regions [13]. In contrast, MBDE relies on the binding of the MBD polypeptide to any of the numerous methylated regions in the genome—extragenic as well as intragenic, and CpG shores and shelves as well as the islands they flank (Fig. 1b-c). Therefore, MBDE allowed us to recover more genomic information than TE.

Our analysis also confirmed that both MBDE and TE preferentially cover CpG-dense regions (Fig. 3b), but the mean O:E CpG ratio of those covered by TE was slightly higher than that of MBDE-covered regions (difference between means = 0.075). This small increase is consistent with the fact that TE detected 25,291 (96%) of the 26,361 “canonical” CpG islands located in non-sex chromosomes, as opposed to only 15,423 (59%) detected with MBDE. Most CpG islands are unmethylated in human tissues [49, 50] and will therefore be missed by the MBD polypeptide, which binds to methylated regions [22]. In contrast, however, the shores and shelves flanking canonical CpG islands are not missed by MBDE since they are usually methylated. Indeed, by considering a broader window that includes shelves and shores, as well as the island they flank (i.e., sSISs), MBDE actually covered 93.5% of these regions (24,644, Fig. 1f).

As for the CpG sites covered by both enrichment technologies, previous comparisons have shown high concordance in the methylation levels identified, in non-colorectal cells or tissues, by MBDE and bisulfite-sequencing approaches (reduced representation bisulfite sequencing [51] and whole genome bisulfite sequencing [52, 53]). Unlike these studies, our work included both diseased tissues (i.e., cancers) and normal colorectal tissues, with the latter as a reference. We could thus focus on methylation level *alterations* in primary CRCs, as compared with basal levels observed in normal colorectal mucosa, and we also assessed the performance of MBDE vs. TE in regions differing in CpG-density (Fig. 3c). On the whole, the methylation changes identified with the two methods were concordant, with correlation values ranging from 0.59 to 0.65 (Fig. 3d). Significant changes in methylation were observed in high-, intermediate, and low-CpG-density regions. Interestingly, however, MBDE identified hypermethylation in high-density regions that was not detected by TE, and TE consistently detected more hypomethylation, regardless of the regions’ CpG density (Fig. 3c-d). The latter difference was likely due to the limited magnitude of the changes involving hypomethylation and their tendency to occur in relatively short stretches of DNA. Both factors reduce the odds that these alterations will be detected by MBDE, which cannot quantify methylation levels at single CpGs [51, 52].

Despite the evidence for concordance between MBDE and TE, the differences (e.g., those related to cost, coverage achieved, and resolution) should also be considered when choosing between the two types of enrichment protocols (see [51, 54] for formal comparisons). In general, for the study of regional methylation, single-CpG resolution might not be necessary, and a lower-cost method of enrichment like MBDE can be used. Targeted enrichment, however, is more suitable when there is a need for CpG resolution of methylation levels (e.g., identification of methylation at transcription-factor binding sites).

## Conclusions

While tumor-associated *hyper*methylation at specific genomic regions—promoters in particular—appears to be a progressive phenomenon that plateaus before CRC cells disseminate to the liver, the progression of CRC-associated *hypo*methylation seems to continue in metastatic lesions, with low-magnitude changes that are nonetheless evident throughout the genome. At this stage, the clinical implications of our findings are unknown. One can reasonably speculate, however, that the relatively fixed nature of the CRC hypermethylome could render liver metastases less “flexible” and consequently more vulnerable to certain medical treatments, whereas the ongoing extension of hypomethylation after liver seeding might lead to the emergence of adaptive changes favoring drug resistance. Further study of this issue, using methods capable of characterizing whole-genome DNA methylation and gene expression, is therefore important since it might well reveal new prospects for the treatment of patients with late-stage CRC.

## Supplementary information

**Additional file 1: Supplementary Fig. 1.** MDS (multidimensional scaling) plot of DNA methylation levels in the 21 samples included in the study. While normal mucosa samples (blue circles) were clustered together and separated from tumors, there is no obvious separation between CRCs (red circles) and liver metastases (green circles).

**Additional file 2: Supplementary Fig. 2.** DMR length distribution. Length in base pairs of hypermethylated and hypomethylated DMRs in: A. the extragenic vs. intragenic genomes; B. among the intragenic genome components; C. the sSISs and extra-sSISs genomic segments; and D. among the sSISs components. NM: normal mucosa, CRC: primary cancer, Met: metastasis.

**Additional file 3: Supplementary Fig. 3.** Integrative Genomics Viewer snapshots showing examples of significantly hyper- or hypomethylated DMRs detected with MBDE, TE, or both enrichment methods. Abbreviations: DMR: differentially methylated region; MBDE: MBD enrichment; TE: targeted enrichment; NM: normal mucosa; CRC: colorectal cancer. Tracks corresponding to NM samples, primary CRCs, and CRC liver metastases are shown in blue, red, and green, respectively. For MBDE, track-landscape heights indicate read depth; for TE, track-bar heights indicate the methylation level (%) at a given CpG site. A: Large, hypermethylated DMR overlapping the CpG island in the *EYA4* promoter, detected by both methods. B: Four consecutive, hypermethylated DMRs detected by MBDE. The fourth one was also detected by TE, with a probe directed specifically at CpG island no. 37 at this genomic locus. C: This DMR was found to be significantly hypermethylated only with MBDE. At the adjusted *P*-value cutoff used in the analysis of TE data, the differential methylation in this region was not significant, although methylation levels at many CpG sites (indicated by bar heights) are clearly higher in primary CRCs than in NM samples. D and E: Examples of hypomethylated DMRs detected only with TE: like most of the hypomethylated DMRs we found, these two are shorter than the hypermethylated DMRs. In general, the hypomethylated DMRs were also characterized by relatively small differences with respect to the methylation levels in NM. (See Results and Discussion.)

**Additional file 4: Supplementary Fig. 4.** In-silico analysis of Gene Expression Omnibus DataSet GSE53051 (submitted by Timp et al., reference [39]). For each sample, the normalized average beta values per probe were downloaded. A. MDS (multidimensional scaling) plot of the beta values for 6 patients with paired normal mucosa (NM), primary cancer (CRC) and liver metastasis (Met). The limma package (reference [25]) was used to identify the 1000 probes displaying the highest variability and to plot the MDS. Consistent with the findings of our own study (Supplementary Fig. 1), normal mucosa samples from the DataSet clustered together and were appreciably separated from tumors, whereas there was no obvious separation between CRCs and liver metastases. B. Scatter plots of the mean beta values per tissue (Met vs NM, CRC vs NM, CRC vs Met) for all the probes (Genome, top 3 plots), and for probes located in CpG Islands (Islands, bottom 3 plots). CRCs and Mets had similar methylomes, both with Genome and Island probes (top and bottom panels on the right, respectively), while skewed profiles towards hypomethylation (Genome probes) or hypermethylation (Islands probes) in tumors (vs NM) were detected (the four panels on the left and in the middle). C. The similarity of the CRC and Met methylation patterns is also reflected by the mean beta values per sample across all probes (Genome, top) and the probes in CpG Islands (Islands, bottom).

**Additional file 5: Supplementary Table 1.**

## Data Availability

The datasets generated and analyzed during the current study are available in the *ArrayExpress* repository (accession numbers: E-MTAB-8232).

## Abbreviations

CpGs: CpG sites
CIMP: CpG island methylator phenotype
CRC: Colorectal cancer
DMRs: Differentially methylated regions
FDR: False discovery rate
logFC: Log-fold change
MBD: methylCpG binding domain
MBDE: MBD protein-mediated enrichment
MSI: Microsatellite instability
O:E CpG ratio: Ratio of the observed to expected numbers of CpGs
sSISs: Shelf-shore-island-shore-shelf
TE: Targeted enrichment
