# Supplementary figures and images for: The DNA hypermethylation phenotype of colorectal cancer liver metastases resembles that of the primary colorectal cancers
Source: BMC Cancer. 2020 Apr 6;20:290. doi: 10.1186/s12885-020-06777-6 (PMC7137338; doi:10.1186/s12885-020-06777-6)

Supplementary Figure 1. Orjuela S. et al.

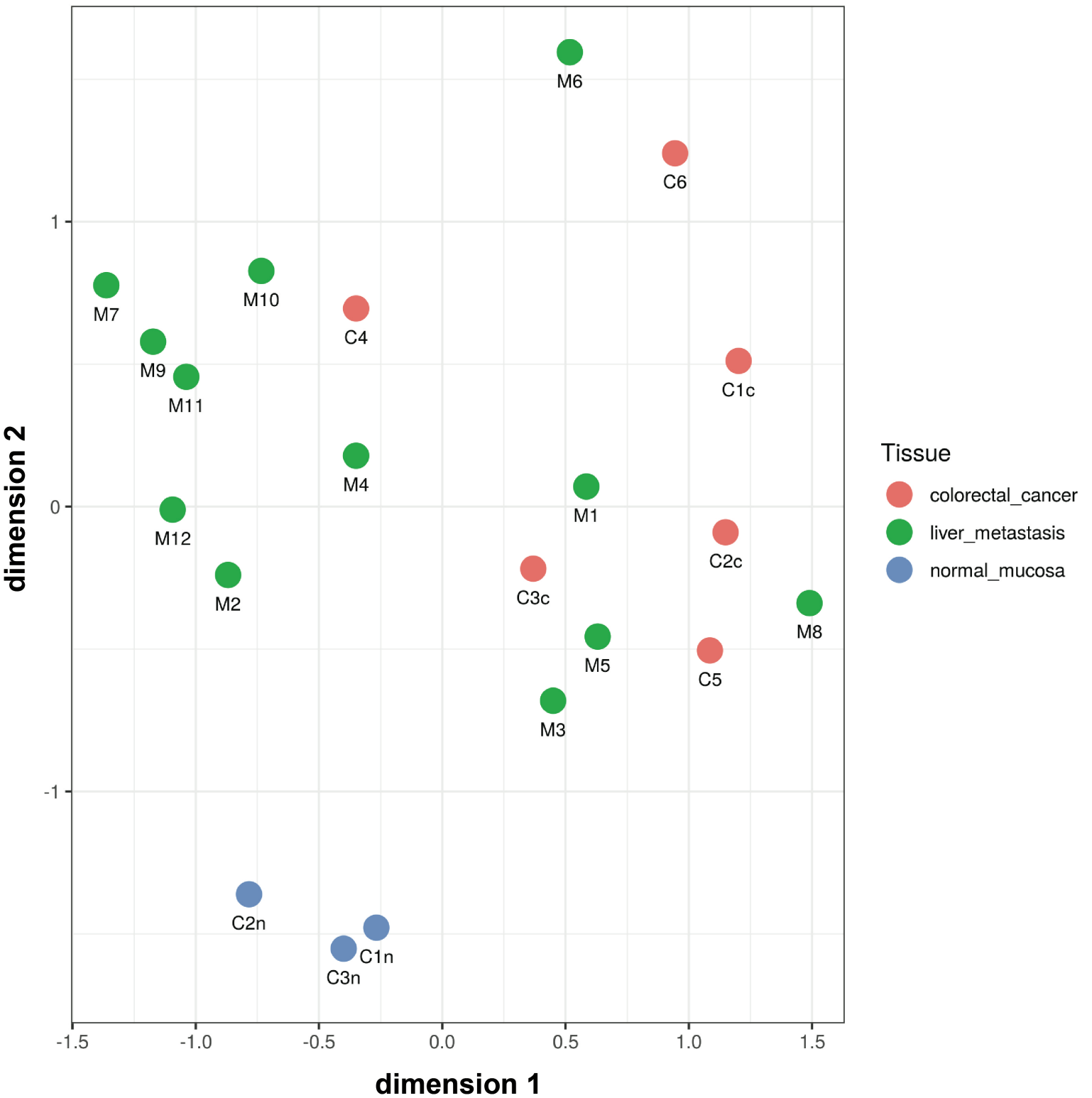

Supplement: Supplementary file 1 — Additional file 1: Supplementary Fig. 1. MDS (multidimensional scaling) plot of DNA methylation levels in the 21 samples included in the study. While normal mucosa samples (blue circles) were clustered together and separated from tumors, there is no obvious separation between CRCs (red circles) and liver metastases (green circles). [file 12885_2020_6777_MOESM1_ESM.pdf]

Supplementary Figure 2. Orjuela S. et al.

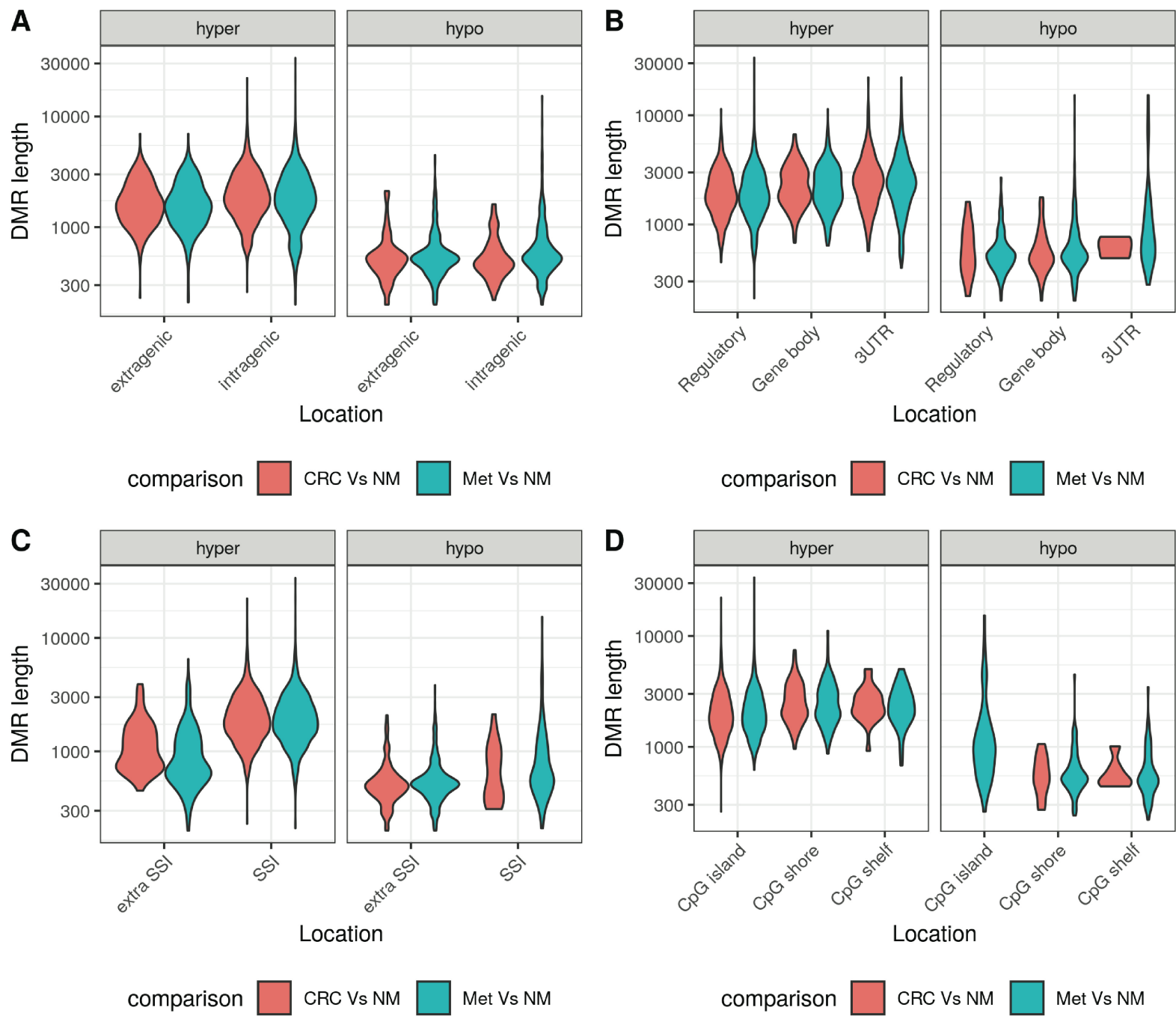

Supplement: Supplementary file 2 — Additional file 2: Supplementary Fig. 2. DMR length distribution. Length in base pairs of hypermethylated and hypomethylated DMRs in: A. the extragenic vs. intragenic genomes; B. among the intragenic genome components; C. the sSISs and extra-sSISs genomic segments; and D. among the sSISs components. NM: normal mucosa, CRC: primary cancer, Met: metastasis. [file 12885_2020_6777_MOESM2_ESM.pdf]

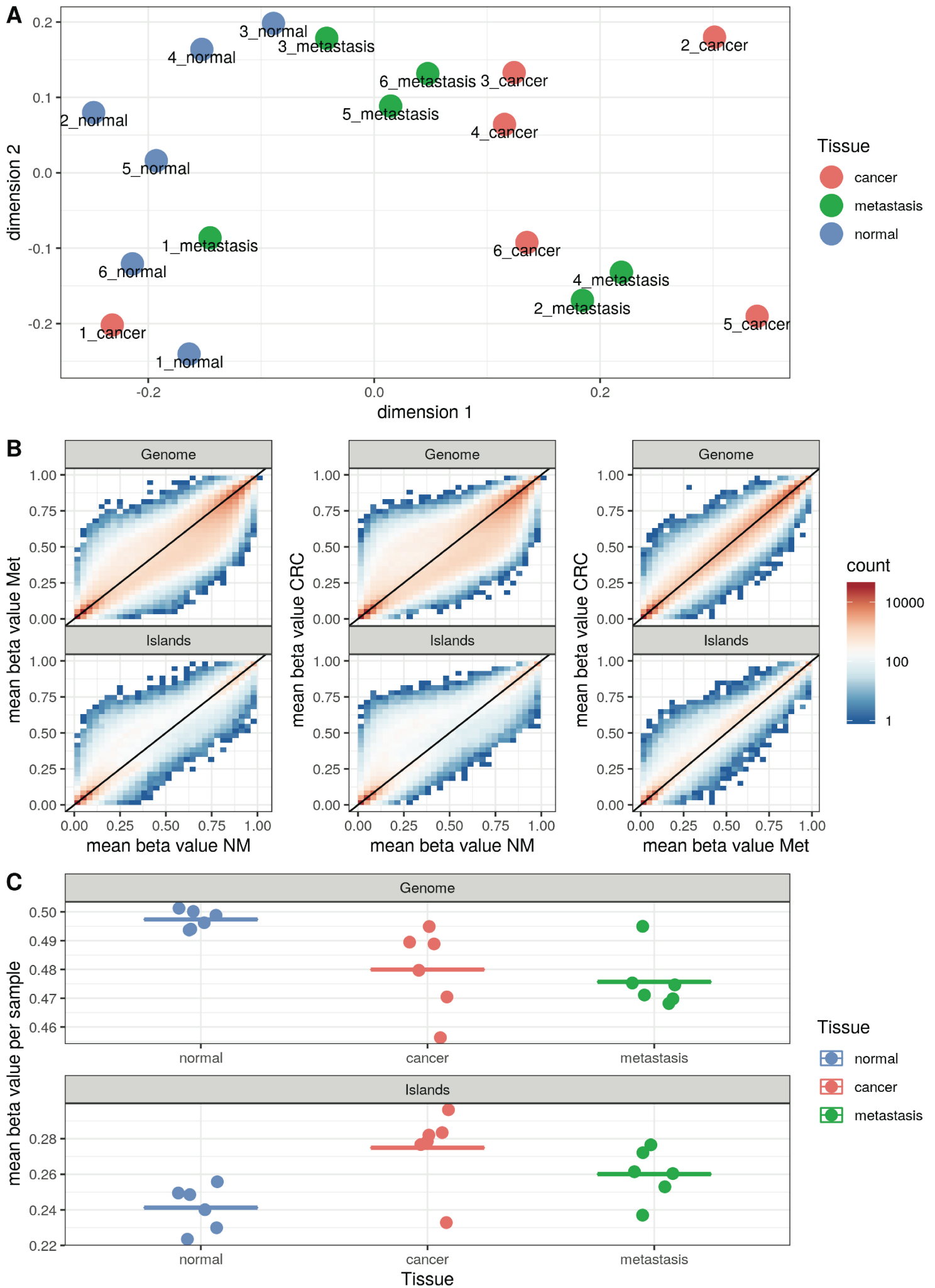

Supplement: Supplementary file 4 — Additional file 4: Supplementary Fig. 4. In-silico analysis of Gene Expression Omnibus DataSet GSE53051 (submitted by Timp et al., reference [39]). For each sample, the normalized average beta values per probe were downloaded. A. MDS (multidimensional scaling) plot of the beta values for 6 patients with paired normal mucosa (NM), primary cancer (CRC) and liver metastasis (Met). The limma package (reference [25]) was used to identify the 1000 probes displaying the highest variability and to plot the MDS. Consistent with the findings of our own study (Supplementary Fig. 1), normal mucosa samples from the DataSet clustered together and were appreciably separated from tumors, whereas there was no obvious separation between CRCs and liver metastases. B. Scatter plots of the mean beta values per tissue (Met vs NM, CRC vs NM, CRC vs Met) for all the probes (Genome, top 3 plots), and for probes located in CpG Islands (Islands, bottom 3 plots). CRCs and Mets had similar methylomes, both with Genome and Island probes (top and bottom panels on the right, respectively), while skewed profiles towards hypomethylation (Genome probes) or hypermethylation (Islands probes) in tumors (vs NM) were detected (the four panels on the left and in the middle). C. The similarity of the CRC and Met methylation patterns is also reflected by the mean beta values per sample across all probes (Genome, top) and the probes in CpG Islands (Islands, bottom). [file 12885_2020_6777_MOESM4_ESM.pdf]
