# Supplementary material for: The DNA hypermethylation phenotype of colorectal cancer liver metastases resembles that of the primary colorectal cancers
Source: BMC Cancer. 2020 Apr 6;20:290. doi: 10.1186/s12885-020-06777-6 (PMC7137338; doi:10.1186/s12885-020-06777-6)

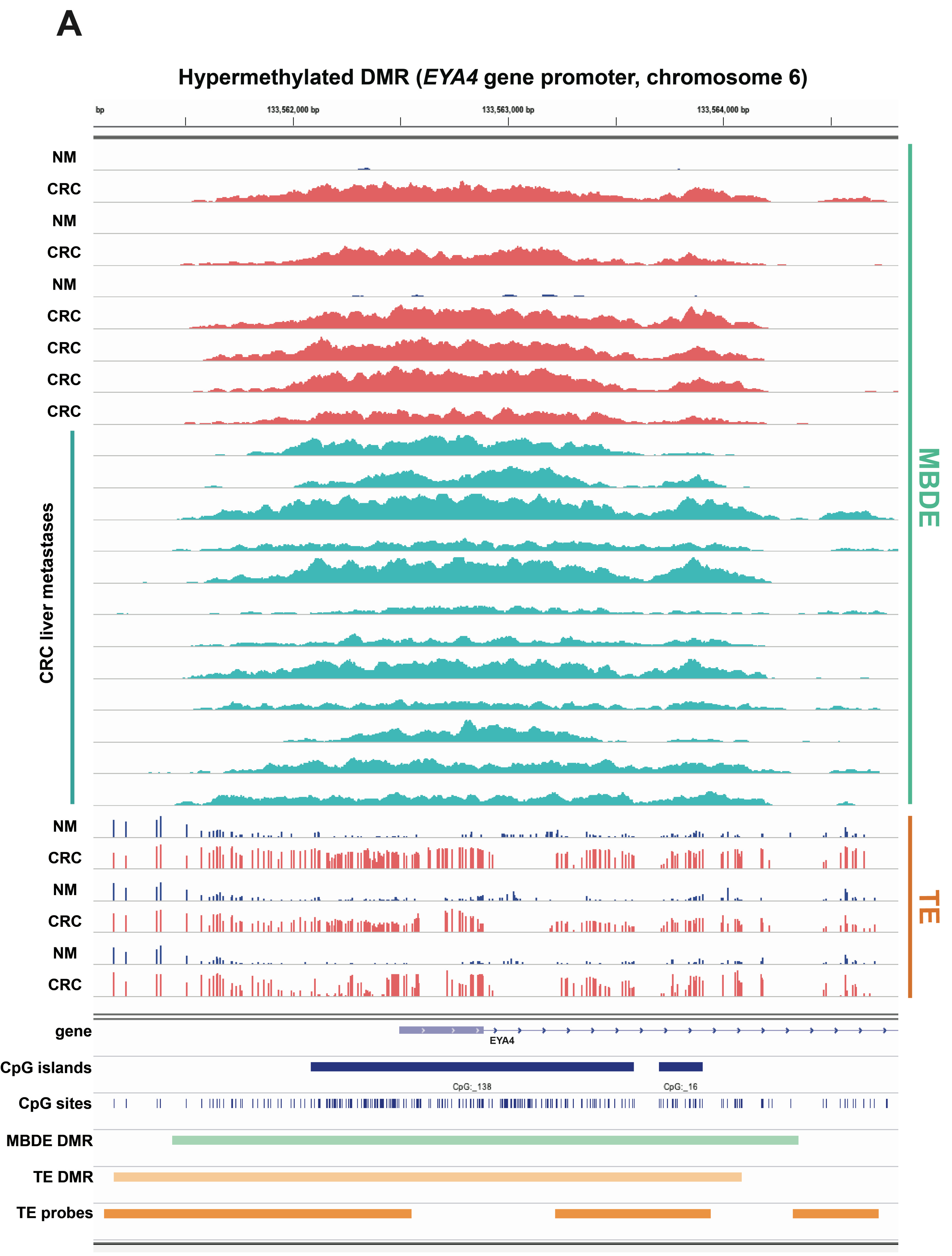
A
Hypermethylated DMR (EYA4 gene promoter, chromosome 6)
bp
133,562,000 bp
133,563,000 bp
133,564,000 bp
NM
CRC
CRC liver metastases
MBDE
TE
gene
EYA4
CpG islands
CpG:_138
CpG:_16
CpG sites
MBDE DMR
TE DMR
TE probes

B

Hypermethylated DMRs (*NBPF19* gene, chromosome 1)

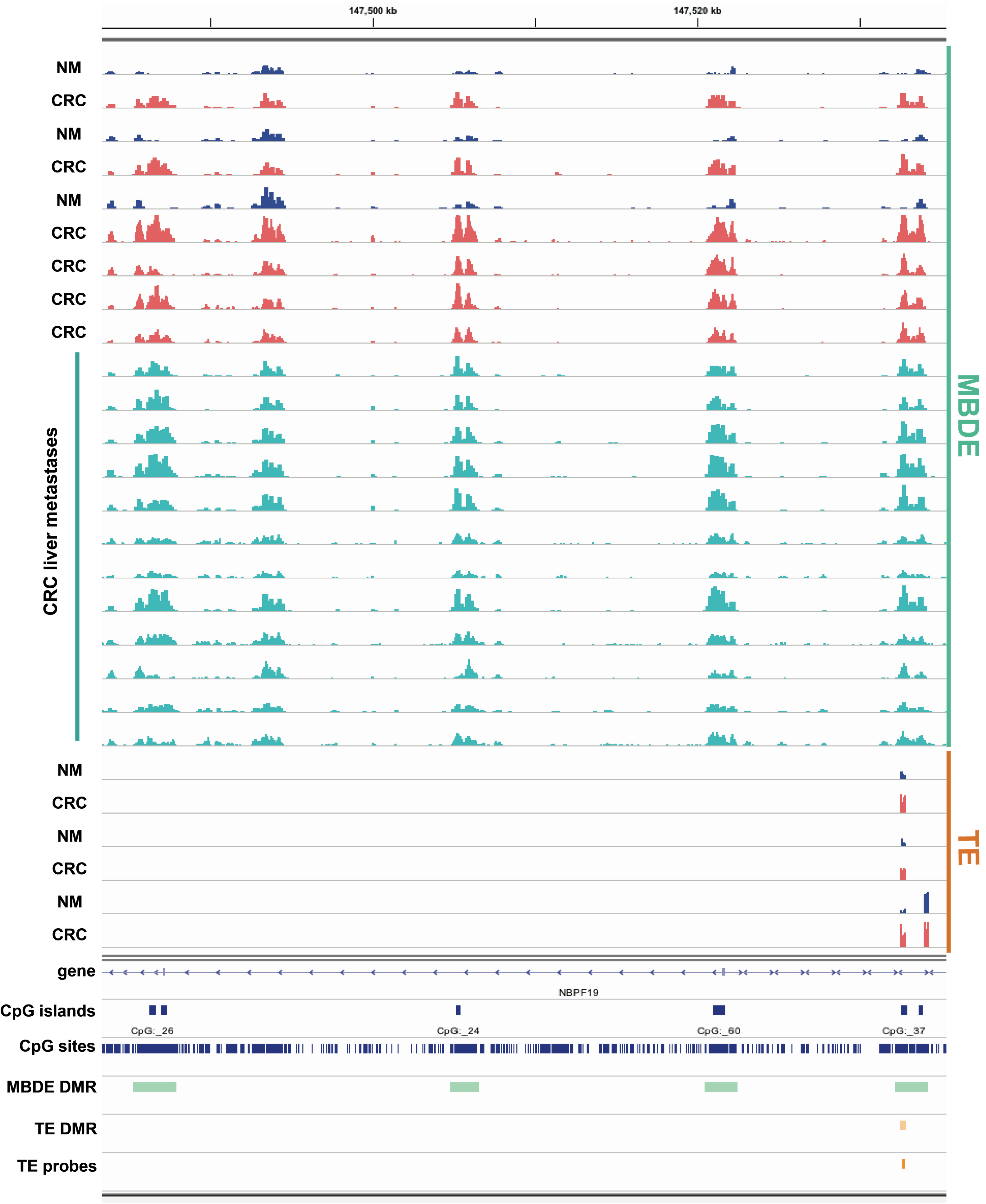

C

Hypermethylated DMR (*MSX1* gene, chromosome 4)

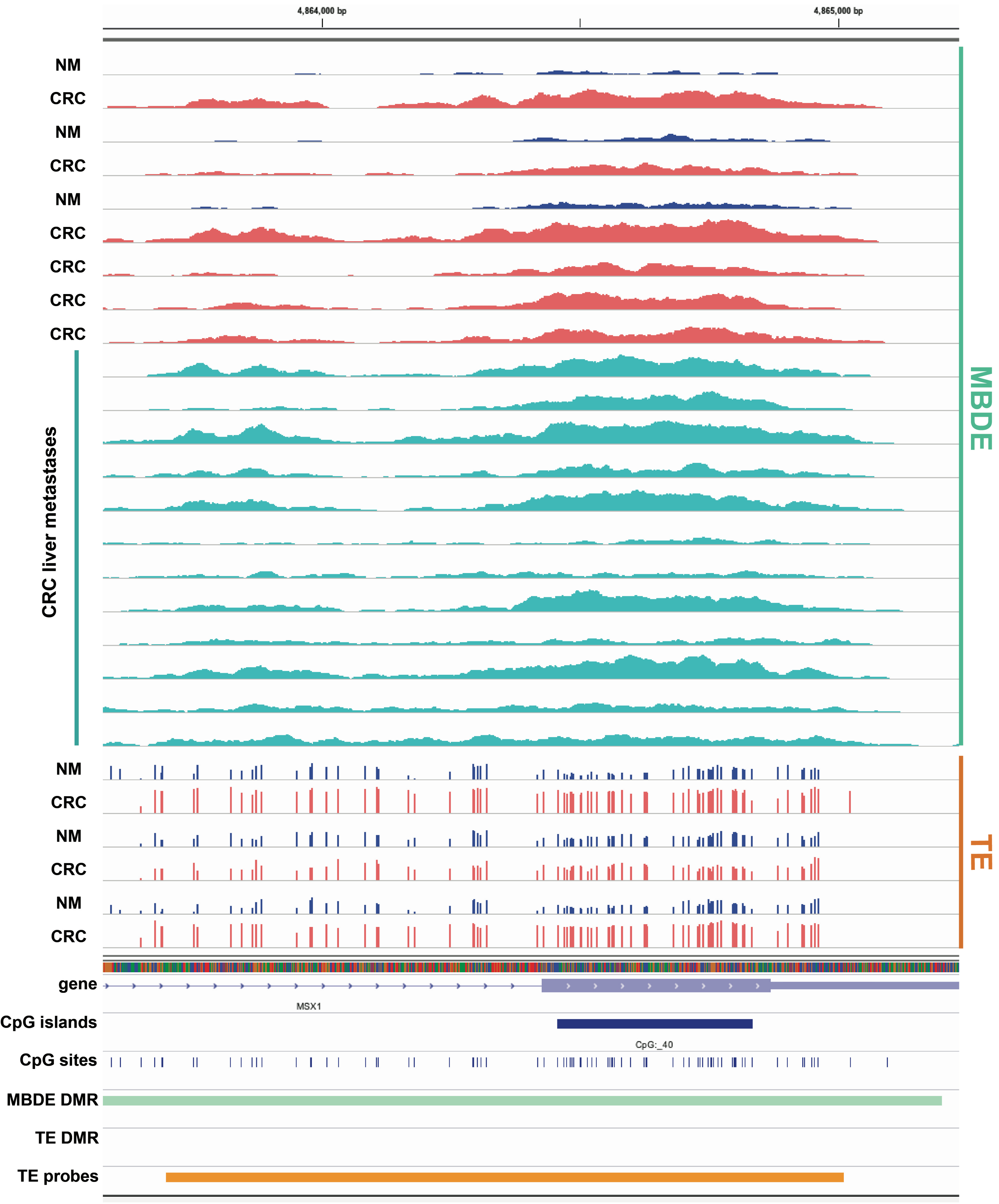

D

Hypomethylated DMR (*WNT2* gene, chromosome 7)

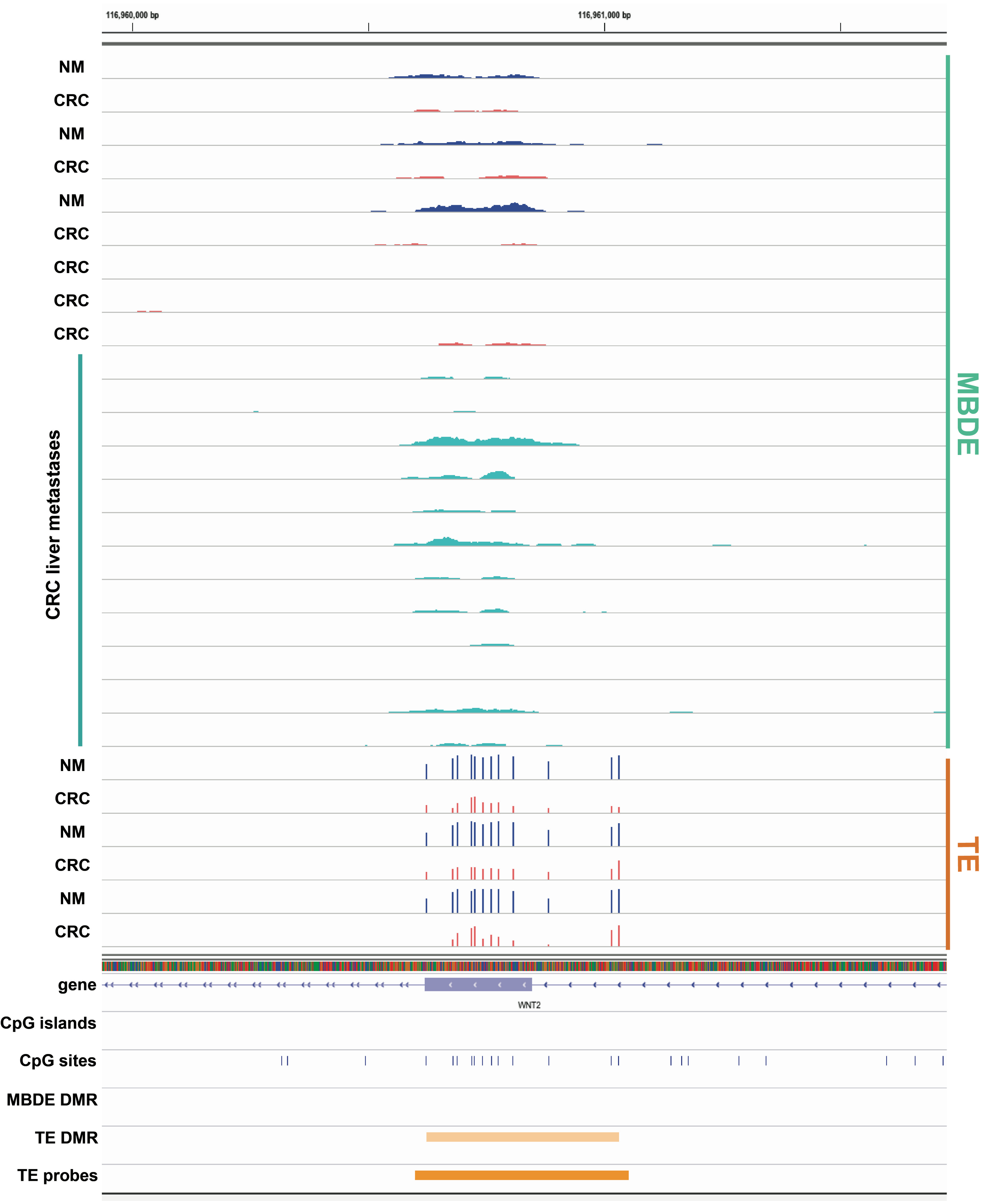

E

Hypomethylated DMR (*SULF1* gene promoter, chromosome 8)

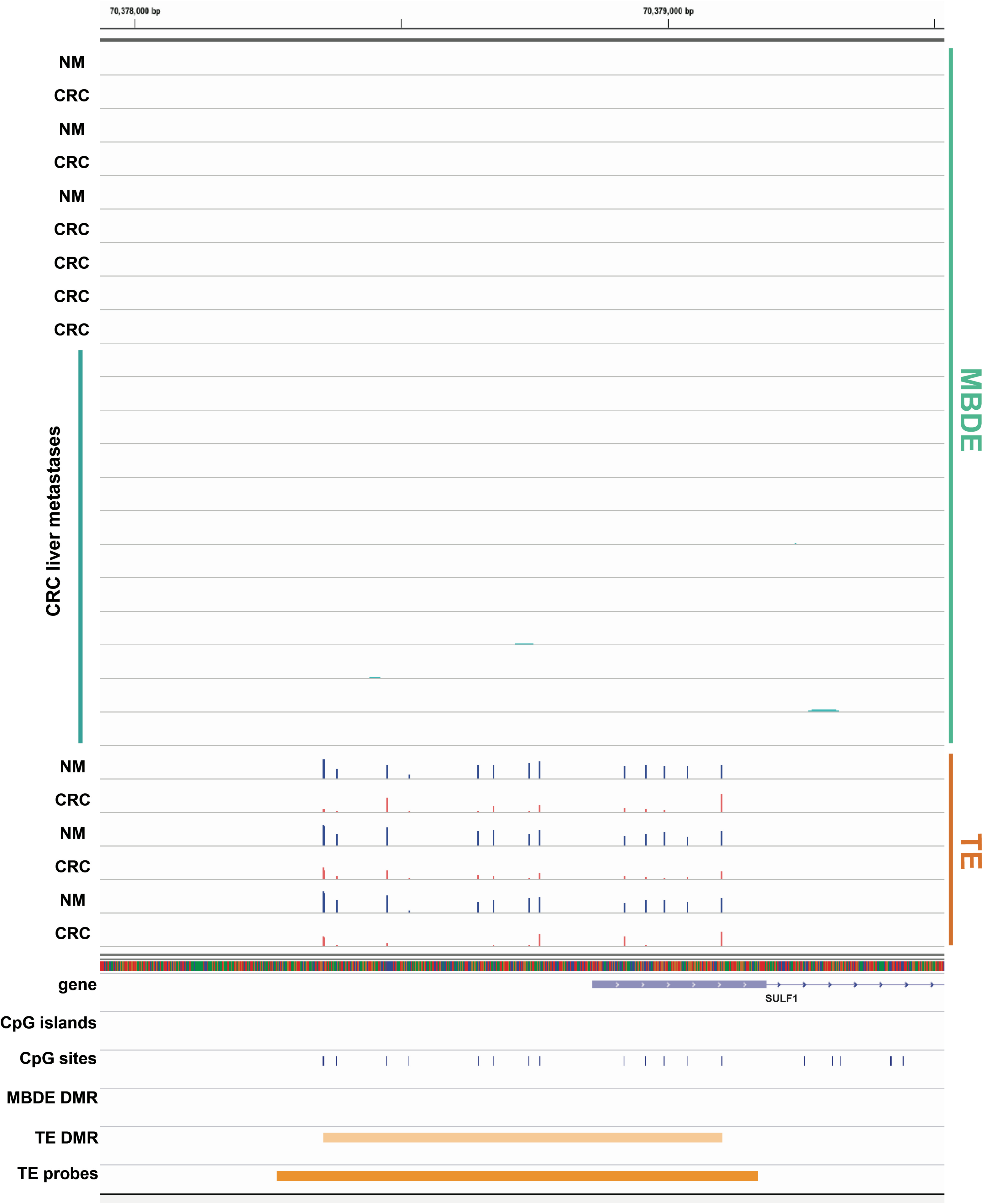

Supplement: Supplementary file 3 — Additional file 3: Supplementary Fig. 3. Integrative Genomics Viewer snapshots showing examples of significantly hyper- or hypomethylated DMRs detected with MBDE, TE, or both enrichment methods. Abbreviations: DMR: differentially methylated region; MBDE: MBD enrichment; TE: targeted enrichment; NM: normal mucosa; CRC: colorectal cancer. Tracks corresponding to NM samples, primary CRCs, and CRC liver metastases are shown in blue, red, and green, respectively. For MBDE, track-landscape heights indicate read depth; for TE, track-bar heights indicate the methylation level (%) at a given CpG site. A: Large, hypermethylated DMR overlapping the CpG island in the EYA4 promoter, detected by both methods. B: Four consecutive, hypermethylated DMRs detected by MBDE. The fourth one was also detected by TE, with a probe directed specifically at CpG island no. 37 at this genomic locus. C: This DMR was found to be significantly hypermethylated only with MBDE. At the adjusted P-value cutoff used in the analysis of TE data, the differential methylation in this region was not significant, although methylation levels at many CpG sites (indicated by bar heights) are clearly higher in primary CRCs than in NM samples. D and E: Examples of hypomethylated DMRs detected only with TE: like most of the hypomethylated DMRs we found, these two are shorter than the hypermethylated DMRs. In general, the hypomethylated DMRs were also characterized by relatively small differences with respect to the methylation levels in NM. (See Results and Discussion.) [file 12885_2020_6777_MOESM3_ESM.pdf]
